# Maternal age at first childbirth and under-five morbidity in sub-Saharan Africa: analysis of cross-sectional data of 32 countries

**DOI:** 10.1186/s13690-021-00674-5

**Published:** 2021-08-24

**Authors:** Bright Opoku Ahinkorah

**Affiliations:** School of Public Health, Faculty of Health, University of Technology Sydney, Ultimo, Australia

**Keywords:** Age at first childbirth, Under-five morbidity, Sub-Saharan Africa, Global Health

## Abstract

**Background:**

The prevalence of childhood morbidity remains high in low-and middle-income countries, including sub-Saharan Africa (SSA). In this study, the association between maternal age at first childbirth and under-five morbidity in SSA was examined.

**Methods:**

This was a cross-sectional study involving nationally-representative data from the most recent Demographic and Health Surveys conducted in 32 countries in SSA from 2010 to 2019. A sample size of 311,603 mothers of children under-five was considered. The outcome variable for this study was under-five morbidity. This variable was derived from the experience of fever, cough, and diarrhoea among children under-five. Both multilevel and binary logistic regression models were used to test the hypothesis that adolescent childbirth is associated with under-five morbidity. The results were presented as crude odds ratios (cORs) and adjusted odds ratios (aORs), with 95 % confidence intervals (CIs).

**Results:**

Children born to mothers whose first childbirth occurred at < 20 years were 16 % times more likely to suffer from under-five morbidity, compared to those whose mothers’ first childbirth occurred at age ≥ 20 years [cOR = 1.16; CI = 1.13–1.19], and this persisted but with reduced odds after controlling for covariates [aOR = 1.10; CI = 1.07–1.12]. At the country level, children born to mothers whose first childbirth occurred at < 20 years were more likely to suffer from under-five morbidity, compared to those whose mothers’ first childbirth occurred at age ≥ 20 years in Angola, Burundi, Congo DR, Guinea, Kenya, and Uganda.

**Conclusions:**

In this study, an association between adolescent childbirth and morbidity in children under five in SSA has been established. The study concludes that under-five morbidity is higher among children born to mothers whose first childbirth occurred before 20 years compared to those whose mothers’ first childbirth occurred at 20 years and above. The findings indicate that in order to reduce under-five morbidity, there is the need to deal with adolescent childbearing through cultural and social change, coupled with engagement of adolescents and stakeholders in adolescent sexual and reproductive health programmes.

## Background

Globally, morbidity in children has been considered as the major cause of death in children under five [1]. According to the World Health Organisation (WHO), infectious diseases, including pneumonia, diarrhoea and malaria, along with pre-term birth, birth asphyxia and trauma, and congenital anomalies remain the leading causes of death for children under five [2]. The prevalence of childhood morbidity remains high in low-and middle-income countries (LMICs), including sub-Saharan Africa (SSA) [3]. For instance, studies have shown that diarrhea and fever are among the major diseases that contribute to the burden of childhood morbidity and mortality in SSA [3–5]. Across LMICs, 10 % of deaths in under-five children is attributable to diarrhoea [6]. A recent study in 31 countries in SSA also found that about 22 % of children in SSA suffered from fever, 23 % suffered from cough and 16 % suffered from diarrhea between 2010 and 2018 [3].

In SSA, numerous studies have established significant associations between several demographic and socio-economic characteristics of women as well as child characteristics and morbidity among children under five. For instance, a number of studies have found associations between maternal age, education, wealth index, employment status, marital status, birth order, child’s age, child’s size at birth and child’s sex as predictors of morbidity among children under five [3, 5, 7]. Specifically, these studies found higher odds of under-five morbidity among older women, women with low level of education, those with poor wealth index, unemployed women, women with higher birth order, younger children, female children and children with small size at birth [3, 5, 7]. One key factor that these studies failed to examine is the role maternal age at first childbirth plays in morbidity among children under five.

Studies that have examined the association between maternal age at first childbirth and morbidity among children have found that children born to mothers whose first childbirth occurred when they were adolescents are the most vulnerable to poor child health outcomes [8, 9]. The possible reasons that have been linked to this association are the low socio-economic status and weak immune systems of adolescent mothers [10, 11]. Moreover, women who give birth as adolescents are less likely to use antenatal, delivery and postnatal care services compared to those who give birth as adults [12, 13]. Furthermore, the fact that such births are more likely to be their first birth, may account for increased risks of under-five morbidity [14].

Studies in SSA have established that the negative consequences of adolescent childbearing on child health may not only be short and medium-term but long term as well [15, 16]. For instance, adolescents who have had a child are more likely to be disadvantaged socio-economically even after several years due to dropping out of school, unemployment and abandonment by parents [16, 17]. Others may also go through long-term psychological problems such as anxiety and depression due to stigmatisation [18–20]. All these may have negative consequences on the health status of their subsequent children who may be born when they are adults.

Despite the established association between maternal age at first childbirth and under-five morbidity, there are few studies on the association between these phenomena in SSA and these were only single country studies [21–23]. In this study, the association between maternal age at first childbirth and under-five morbidity in 32 countries in SSA was examined. Findings from the study can raise awareness and show the need to have adequate policies and programmes to deal with adolescent childbearing and child morbidity in SSA.

## Methods

### Study design

This study was based on a cross-sectional survey from the Demographic and Health Surveys (DHS) of 32 countries in SSA. In this study, the children’s files, which contain data on children under-five of women aged 15–49 were used. The DHS data is often gathered every 5 years, with longer periods in some countries due to contextual factors.

### Sampling and data collection procedure

A two-stage sampling procedure is followed in gathering data for the DHS. The two stages involve the selection of clusters usually called enumeration areas (EAs) at the first stage and the selection of households for the survey at the second stage. Detailed description of the sampling methodology and data collection processes are published elsewhere [24]. The inclusion criteria for considering a DHS in this study is that it has to be published between 2010 and 2019, should have information on age at first childbirth, under-five morbidity and all other important variables considered in this study. Using these criteria, the DHS datasets of 32 countries in SSA with a sample size of 311,603 were considered. The countries included in this study and their samples are shown in Table 1. The manuscript was prepared in line with the Strengthening Reporting of Observational studies in Epidemiology (STROBE) reporting guidelines [25].

**Table 1:** Distribution of study sample by country

Survey Countries	Survey Year	Weighted Sample	Weighted Percentage
Angola	2016	12,539	4.02
Benin	2018	12,507	4.01
Burkina Faso	2010	13,994	4.49
Burundi	2017	12,405	3.98
Cameroon	2018	9441	3.03
Chad	2015	16,499	5.30
Comoros	2012	2968	0.95
Congo	2011-12	7680	2.46
Congo DR	2013-14	16,780	5.38
Cote D’lvoire	2011-12	6588	2.11
Ethiopia	2016	10,340	3.32
Gabon	2012	4536	1.46
Gambia	2013	7436	2.39
Ghana	2014	5421	1.74
Guinea	2018	7232	2.32
Kenya	2014	8855	2.84
Lesotho	2014	2812	0.91
Liberia	2013	5869	1.88
Malawi	2016	16,284	5.23
Mali	2018	9549	3.06
Namibia	2013	4140	1.33
Niger	2012	12,057	3.87
Nigeria	2018	30,677	9.84
Rwanda	2015	7589	2.44
Senegal	2010-11	10,724	3.44
Sierra Leone	2019	8809	2.83
South Africa	2016	3248	1.04
Tanzania	2016	9217	2.96
Togo	2013-14	6261	2.01
Uganda	2016	14,111	4.53
Zambia	2018	9199	2.95
Zimbabwe	2015	5836	1.87

### Study variables

#### Outcome variable

The outcome variable for this study was under-five morbidity. This variable was derived from the experience of fever, cough and diarrhoea among children under-five. For each of these morbid conditions, women were asked if their children had suffered from them at any time in the 2 weeks preceding the survey. The responses for each of the questions were “Yes” and “No”. Children who suffered from at least one of these morbid conditions were considered as those who had under-five morbidity and those who did not suffer from any of these conditions were considered as those who had never experienced under-five morbidity.

#### Explanatory variables

Age at first childbirth categorised into < 20 years (adolescent childbirth) and ≥ 20 years (adult childbirth) was the key explanatory variable in this study. This variable was derived from the question, “how old were you when you first gave birth to [name]?” The responses to this question were in single years. Based on the findings of previous studies on under-five morbidity [3, 21, 26, 27], mother’s age, marital status, pregnancy intention, place of residence, mother’s education level, wealth quintile, sex of child, child’s weight, number of antenatal care [ANC] visits, place of delivery, and assistant during delivery were considered as covariates.

### Statistical analysis

In testing the hypothesis that children under-five born to women whose first childbirth occurred before 20 years are more likely to suffer from either fever, cough or diarrhea, several statistical analyses were carried out using Stata version 14.0. First, bar charts were used to show the prevalence of adolescent childbirth, under-five morbidity and the distribution of under-five morbidity across age at first childbirth of women. Next, using chi-square test, the association between age at first childbirth and fever, cough and diarrhea for each of the 32 countries in SSA were presented using a table. Thirdly, to account for the hierarchical structure and the clustering effect of the datasets, multilevel binary logistic regression models were used to show the association between age at first childbirth and under-five morbidity while controlling for the covariates. Model 0 showed the variance in under-five morbidity attributed to the clustering of the primary sampling units (PSUs) without any explanatory variable. Model I contained the age at first childbirth and under-five morbidity. Model II had age at first childbirth and under-five morbidity while controlling for all the covariates. The Stata command “melogit” was used in fitting these models. The log-likelihood and Akaike’s Information Criterion (AIC) tests were used to check for model fitness. Finally, both bivariate and multivariable binary logistic regression models were used to test the hypothesis that adolescent childbirth is associated with under-five morbidity in each of the countries. The results were presented as crude odds ratios (cORs) and adjusted odds ratios (aORs), at 95 % confidence intervals (CIs). Sample weights were applied using the variable v005 and the survey command in Stata was used to adjust for the complex sampling structure of the data in the regression analyses.

## Results

### Proportion of mothers whose first childbirth occurred when they were adolescents in sub-Saharan Africa

**Fig. 1:**
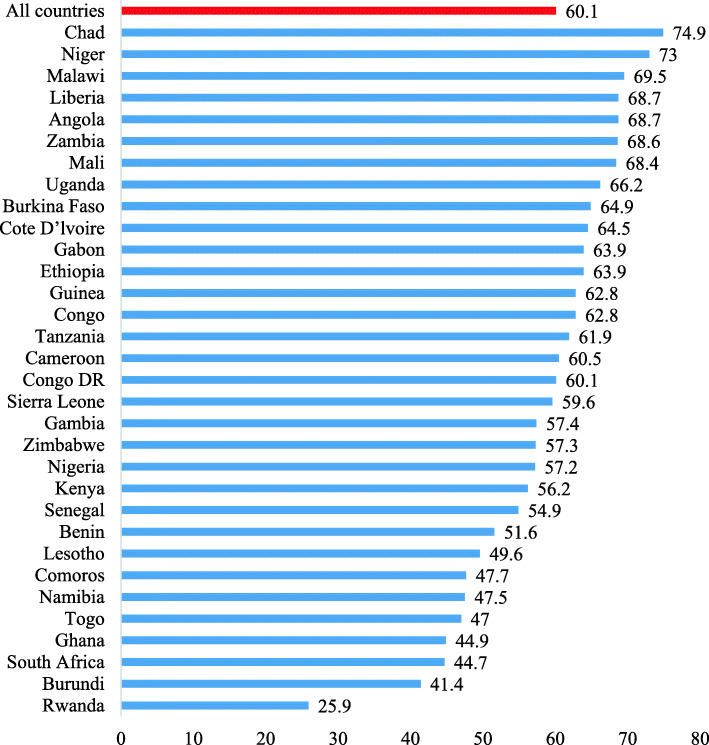
Bar chart showing the proportion of mothers whose first childbirth occurred when they were adolescents in sub-Saharan Africa

In the 32 countries in SSA, the proportion of mothers whose first childbirth occurred when they were adolescents was 60.1%. The highest prevalence was found in Chad (74.9%) and the lowest prevalence was in Rwanda (25.9%) (Fig. 1).

### Prevalence of under-five morbidity in sub-Saharan Africa

**Fig. 2:**
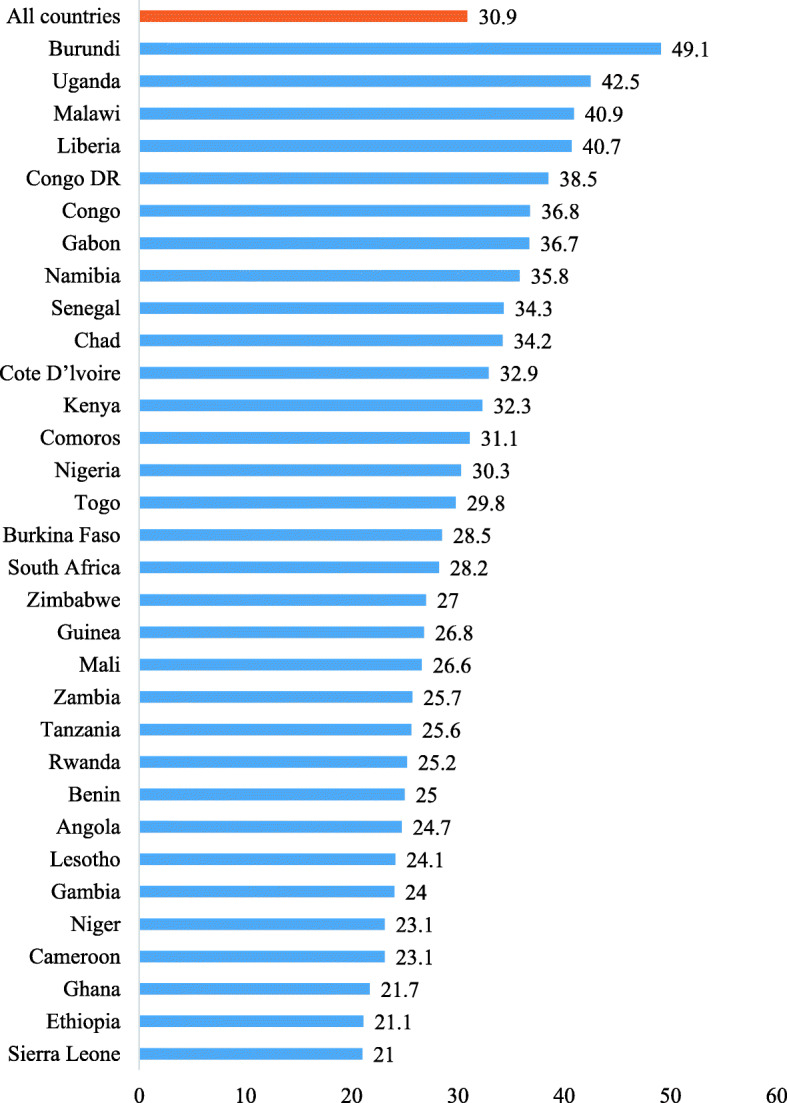
Bar chart showing prevalence of under-five morbidity in sub-Saharan Africa by country

The prevalence of morbidity among children under five in the 32 countries in SSA was 30.9%. Children under-five born to women in Burundi had the highest prevalence of 49.1% while the lowest prevalence of under-five morbidity was found in Sierra Leone (21.0%) (Fig. 2).

### Distribution of the experience of diarrhea, fever, cough, and under-five morbidity accross age at first childbirth

**Fig. 3:**
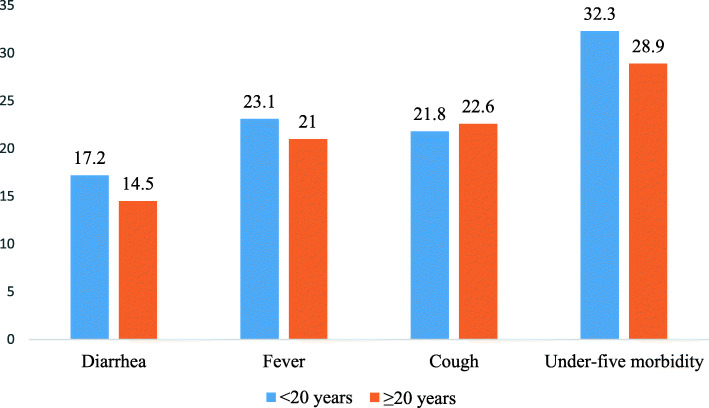
Bar chart showing the distribution of under-five morbidity accross age at first childbirth

Compared to children born to women whose first childbirth occurred when they were adults (14.5%), those born to women whose first childbirth occurred when they were adolescents had the highest prevalence of diarrhea (17.2%). The experience of fever was also higher among children whose mothers’ first childbirth occurred when they were adolescents (23.1%), compared to adults (21%). This was also true of children’s experience of under-five morbidity (32.3% versus 28.9%). On the contrary, the prevalence of cough among children under-five was higher among children whose mothers’ first childbirth occurred when they were adults (22.6%) than adolescents (21.8%) (Fig. 3).

### Age at first childbirth and under-five morbidity by country

Table 2 shows the distribution of under-five morbidity by country. In general, under-five morbidity was significantly higher among children born to mothers whose first childbirth occurred before 20 years compared to 20 years or more in Angola, Burundi, Cameroon, Congo DR, Gabon, Guinea, Kenya, Malawi, Mali, Namibia, Nigeria, Sierra Leone, Togo, Uganda, and Zimbabwe at *p* < 0.05.

**Table 2:** Distribution of under-five morbidity across age at first childbirth by country

Countries	Diarrhea			Fever			Cough			Under-five morbidity		
	< 20 years	≥ 20 years	*p*-values	< 20 years	≥ 20 years	*p*-values	< 20 years	≥ 20 years	*p*-values	< 20 years	≥ 20 years	*p*-values
Angola	16.7	13.5	0.002	16.1	11.9	< 0.001	12.8	11.6	0.210	26.4	21.0	< 0.001
Benin	11.2	9.8	0.015	20.0	18.8	0.186	16.2	16.5	0.709	25.9	24.1	0.056
Burkina Faso	14.9	14.9	0.920	20.1	21.9	0.029	9.8	11.2	0.021	28.1	29.4	0.147
Burundi	24.5	21.0	< 0.001	42.2	37.5	< 0.001	38.1	38.2	0.941	52.3	46.8	< 0.001
Cameroon	13.0	10.7	0.012	16.9	13.6	0.002	17.8	19.1	0.239	24.8	20.4	< 0.001
Chad	22.7	21.6	0.352	24.4	23.7	0.553	20.4	19.7	0.529	34.3	33.7	0.653
Comoros	18.1	16.4	0.323	22.0	22.1	0.954	19.1	18.3	0.665	31.4	30.9	0.803
Congo	20.0	18.2	0.122	26.0	24.1	0.130	28.0	27.8	0.683	37.8	35.1	0.104
Congo DR	18.2	15.3	0.001	31.1	28.2	0.003	32.6	29.9	0.018	40.4	35.8	< 0.001
Cote D’lvoire	18.7	18.4	0.822	23.4	25.8	0.141	21.1	24.2	0.049	32.3	33.9	0.363
Ethiopia	11.9	11.6	0.773	14.3	14.7	0.747	20.3	19.3	0.419	21.0	21.1	0.965
Gabon	18.3	14.1	0.003	26.0	23.6	0.273	42.1	38.9	0.080	36.6	31.3	0.006
Gambia	18.3	17.0	0.347	12.4	11.5	0.862	13.3	14.4	0.028	24.8	23.0	0.295
Ghana	12.2	11.7	0.654	14.3	13.7	0.589	14.5	13.7	0.517	22.3	21.2	0.479
Guinea	15.4	13.2	0.046	17.9	16.1	0.089	11.6	11.0	0.511	28.3	24.2	0.003
Kenya	16.6	13.4	0.003	25.9	21.5	< 0.001	37.8	35.9	0.167	35.2	28.6	< 0.001
Lesotho	13.0	11.4	0.361	16.0	15.0	0.548	30.8	28.7	0.325	25.1	23.2	0.378
Liberia	23.9	20.4	0.040	30.0	28.4	0.417	27.6	22.0	0.002	41.8	38.3	0.099
Malawi	22.5	20.7	0.062	30.1	27.2	0.001	25.2	24.3	0.426	42.0	38.3	< 0.001
Mali	18.0	15.5	0.020	16.2	15.7	0.567	10.3	10.0	0.740	27.3	24.9	0.042
Namibia	22.1	16.9	< 0.001	28.7	24.4	0.016	36.4	29.8	< 0.001	39.1	32.9	0.001
Niger	14.5	13.9	0.524	14.3	15.1	0.472	14.1	15.2	0.313	22.9	23.5	0.613
Nigeria	15.8	9.0	< 0.001	28.2	19.3	< 0.001	14.5	15.2	< 0.001	35.0	24.0	< 0.001
Rwanda	13.1	12.0	0.239	19.9	18.6	0.284	26.0	26.8	0.539	26.1	24.9	0.350
Senegal	21.3	21.0	0.485	21.6	25.1	0.010	19.4	23.4	0.001	33.0	36.0	0.106
Sierra Leone	7.97	6.16	0.020	7.8	15.5	0.018	14.1	13.8	0.702	22.5	18.8	< 0.001
South Africa	11.9	10.0	0.162	21.9	20.2	0.358	27.0	25.5	0.472	29.7	27.0	0.196
Tanzania	12.4	11.7	0.437	19.1	17.2	0.076	15.9	17.2	0.197	26.2	24.8	0.239
Togo	16.7	14.0	0.014	23.9	20.1	0.002	27.5	28.2	0.630	31.8	22.0	0.007
Uganda	20.7	18.7	0.016	36.6	28.7	< 0.001	41.2	41.9	0.599	44.8	38.1	< 0.001
Zambia	16.1	14.0	0.057	16.5	15.1	0.172	22.1	20.4	0.278	26.5	24.1	0.091
Zimbabwe	18.6	14.8	0.002	14.2	13.8	0.763	40.0	38.2	0.231	28.7	24.8	0.018

### Association between adolescent childbirth and under-five morbidity

As shown in Table 3, there was a significant independent association between age at first childbirth and under-five morbidity, with children born to mothers whose first childbirth occurred at < 20 years, 16 % times more likely to suffer from under-five morbidity, compared to those whose mothers’ first childbirth occurred at age ≥ 20 years [cOR = 1.16; CI = 1.13–1.19], and this persisted but with reduced odds after controlling for the covariates [aOR = 1.10; CI = 1.07–1.12] (Model I and Model II of Table 3). At the country level, children born to mothers whose first childbirth occurred at < 20 years, were more likely to suffer from under-five morbidity, compared to those whose mothers’ first childbirth occurred at age ≥ 20 years in Angola, Burundi, Congo DR, Guinea, Kenya, and Uganda (Model II of Table 4).

**Table 3:** Multilevel logistic regression on the association between adolescent childbirth and under-five morbidity in sub-Saharan Africa

Variables	Model 0	Model I cOR (95 % CI)	Model II aOR (95 % CI)
**Fixed effects**			
** Age at first childbirth**			
< 20 years		1.16***[1.13–1.19]	1.10** (1.07–1.12)
≥ 20 years		Reference	Reference
** Random effects**			
Primary sampling unit variance (95 % CI)	0.11 (0.09–0.15)	0.11 (0.08–0.14)	0.06 (0.05–0.08)
Intraclass correlation coefficient	0.03	0.03	0.02
Wald chi-square	Reference	170.27^***^	1430.54^***^
** Model fitness**			
Log-likelihood	-189072.57	-188907.15	-137588.17
Akaike’s Information Criterion	378149.1	377820.3	275226.3
Sample size	311,603	311,603	311,603
Number of clusters	1610	1610	1610

NB: Model II adjusted for mother’s age, marital status, pregnancy intention, place of residence, mother’s education level, wealth quintile, sex of child, child’s weight, number of ANC visits, place of delivery, and assistant during delivery; cOR=crude odds ratio; aOR=adjusted odds ratio

^*^p < 0.05; ^**^p < 0.01; ^***^p < 0.001

**Table 4:** Binary logistic regression on the association between adolescent childbirth and under-five morbidity disaggregated by country

Countries	Model I cOR (95 % CI)	Model II aOR (95 % CI)
Angola	1.43***(1.31–1.57)	1.23*** (1.10–1.39)
Benin	1.09* (1.01–1.18)	1.01 (0.92–1.13)
Burkina Faso	0.95 (0.88–1.03)	0.97 (0.89–1.07)
Burundi	1.25***(1.16–1.35)	1.13* (1.02–1.25)
Cameroon	1.27*** (1.15–1.41)	1.28 (1.12–1.46)
Chad	1.05 (0.97–1.13)	1.03 (0.93–1.14)
Comoros	1.05 (0.90–1.23)	0.89 (0.72–1.11)
Congo	1.08 (0.94–1.19)	1.02 (0.91–1.15)
Congo DR	1.22*** (1.14–1.30)	1.19***(1.09–1.29)
Cote D’lvoire	1.03 (0.93–1.15)	0.98 (0.68–1.46)
Ethiopia	1.07 (0.97–1.19)	1.05 (0.93–1.19)
Gabon	1.20**(1.05–1.36)	1.07 (0.91–1.25)
Gambia	1.06 (0.95–1.18)	1.10 (0.96–1.26)
Ghana	1.05 (0.92–1.19)	0.99 (0.85–1.16)
Guinea	1.19**(1.06–1.33)	1.17* (1.03–1.35)
Kenya	1.26*** (1.15–1.38)	1.13* (1.01–1.26)
Lesotho	1.05 (0.88–1.25)	0.97 (0.78–1.20)
Liberia	1.07 (0.96–1.19)	1.07 (0.94–1.22)
Malawi	1.17***(1.09–1.26)	1.01 (0.93–1.10)
Mali	1.10 (1.00-1.22)	1.04 (0.92–1.18)
Namibia	1.25***(1.11–1.42)	1.05 (0.90–1.23)
Niger	0.97 (0.88–1.06)	0.95 (0.84–1.08)
Nigeria	1.62***(1.54–1.71)	0.97 (0.84–1.13)
Rwanda	1.07 (0.95–1.21)	1.01 (0.58–1.74)
Senegal	0.94 (0.86–1.01)	1.14 (0.77–1.69)
Sierra Leone	1.32***(1.19–1.47)	1.33 (1.17–1.51)
South Africa	1.13 (0.97–1.33)	1.09 (0.91–1.31)
Tanzania	1.06 (0.97–1.17)	1.02 (0.90–1.15)
Togo	1.22***(1.10–1.36)	1.03 (0.90–1.17)
Uganda	1.31***(1.22–1.41)	1.14** (1.04–1.25)
Zambia	1.12*(1.01–1.24)	1.02 (0.90–1.15)
Zimbabwe	1.21**(1.07–1.37)	1.10 (0.95–1.27)

NB: Model II adjusted for mother’s age, marital status, pregnancy intention, place of residence, mother’s education level, wealth quintile, sex of child, child’s weight, number of ANC visits, place of delivery, and assistant during delivery; cOR=crude odds ratio; aOR=adjusted odds ratio

^*^p < 0.05

^**^p < 0.01

^***^p < 0.001

## Discussion

In this study, the hypothesis that children born to mothers whose first childbirth birth occurred before 20 years were more likely to experience under-five morbidity (diarrhea, fever and cough), compared to those whose mothers’ first childbirth occurred at 20 years and above was tested. Findings from the study showed that the risk of under-five morbidity is high among children born to mothers whose first childbirth occurred before 20 years, compared to those whose mothers’ first childbirth occurred at 20 years and above. This finding is consistent with the findings of previous studies in Ethiopia [21, 22] and 55 low-and middle-income countries [8]. Several reasons may account for this finding including poor living conditions among adolescent mothers, no formal education, and low utilization of maternal and child health services [10–13]. These factors explain the overall low socio-economic status and healthcare seeking behaviours of adolescent mothers and determine the likelihood of under-five morbidity [28–32].

Considering that some of the women whose first childbirth occurred when they were adolescents were not adolescents at the time of the survey, the results of the current study on the association between adolescent childbearing and under-five morbidity suggest that the negative effects of adolescent childbearing on under-five morbidity may extend over several years. Hence, the problem is even more profound than we imagine and is not only short or medium-term but long term as well. For instance, adolescents who have had a child are more likely to have low socio-economic status even after several years due to dropping out of school, unemployment and abandonment by parents [16, 17] while others may also go through long-term psychological problems such as anxiety and depression due to stigmatisation [18–20], which may result in under-five morbidity among their subsequent children.

In this study, Angola, Burundi, Congo DR, Guinea, Kenya, and Uganda were the countries where under-five morbidity was higher among children born to mothers whose first childbirth occurred before 20 years compared to those whose mothers’ first childbirth occurred at 20 years and above. This finding is expected because all of these countries have gone through some years of political or civil crises which consequently affected the living conditions in the countries. For instance, the finding that under-five morbidity is higher in children born to mothers whose first childbirth occurred before 20 years, compared to those whose mothers’ first childbirth occurred at 20 years and above in Burundi has been found to be attributed to poor living conditions in the country such as overcrowding and poor housing conditions, inadequate sanitation and unsafe water, where less than 50 % of the population have access to potable water [33]. Other studies have attributed the high childhood morbidity among adolescent mothers in the country to political instability and violent conflict, weakened delivery systems, lower coverage of interventions, disempowering policies and gaps in the continuum of care [23, 34, 35]. Children born to adolescent mothers in Angola, Congo DR, Guinea, Kenya, and Uganda are more likely to experience under-five morbidity due to similar conditions that exist in Burundi since these conditions are more prevalent in countries that have gone through political or civil crisis. These findings imply that in order to reduce under-five morbidity, there is the need to improve the living conditions of mothers as well as children through the implementation of effective sanitation conditions and enhanced access to healthcare for adolescent mothers. In countries where significant associations were not found between adolescent childbearing and under-five morbidity, other maternal characteristics such as mother’s age, marital status, pregnancy intention, place of residence, mother’s education level, and wealth quintile; child characteristics such as sex of child, and child’s weight; and access and use of maternal healthcare services such as number of ANC visits, place of delivery, and assistant during delivery may be responsible for the under-five morbidity [3, 21, 26, 27]. Another possible reason for the lack of association between adolescent childbearing and under-five morbidity could be the existence of protecting factors such as better access to health care in general, a better system of educational and professional rehabilitation for adolescent mothers in those countries [36, 37].

### Strengths and limitations

The major strength of this study is the use of nationally-representative datasets of 32 countries in SSA and the large sample size that made it possible to use high level statistical analyses. Despite this strength, there are some limitations that need to be mentioned. First, the design employed in the DHS is cross-sectional and hence, causal interpretations of the findings cannot be established. Second, age at first childbirth was self-reported, and as a result, there is the possibility of under-and over-reporting of data [38–40].

## Conclusions

In this study, an association between adolescent childbirth and morbidity in children under five in SSA has been established. However, this association was statistically significant in Angola, Burundi, Congo DR, Guinea, Kenya, and Uganda. The study concludes that under-five morbidity is higher among children born to mothers whose first childbirth birth occurred before 20 years compared to those whose mothers’ first childbirth occurred at 20 years and above. The findings indicate that in order to reduce under-five morbidity there is the need to deal with adolescent childbearing through cultural and social change, coupled with engagement of adolescents and stakeholders in adolescent sexual and reproductive health programmes. There is the need for future research to examine the healthcare seeking behaviour for childhood illness among women whose first birth occurred when they were adolescents.

## Data Availability

Data for this study is available at: http://dhsprogram.com/data/available-datasets.cfm.

## Abbreviations

aOR: Adjusted odds ratio
cOR: Crude odds ratio
DHS: Demographic and Health Survey
LMICs: Low-and middle-income countries
SSA: Sub-Saharan Africa
WHO: World Health Organisation
